# A Guide to Elucidate the Hidden Multicomponent Layered Structure of Plant Cuticles by Raman Imaging

**DOI:** 10.3389/fpls.2021.793330

**Published:** 2021-12-17

**Authors:** Peter Bock, Martin Felhofer, Konrad Mayer, Notburga Gierlinger

**Affiliations:** Department of Nanobiotechnology, Institute of Biophysics, University of Natural Resources and Life Sciences, Vienna, Austria

**Keywords:** plant surfaces, waxes, cutin, chalcones, flavonoids, multivariate data analysis, epidermal cell wall, pectin

## Abstract

The cuticle covers almost all plant organs as the outermost layer and serves as a transpiration barrier, sunscreen, and first line of defense against pathogens. Waxes, fatty acids, and aromatic components build chemically and structurally diverse layers with different functionality. So far, electron microscopy has elucidated structure, while isolation, extraction, and analysis procedures have revealed chemistry. With this method paper, we close the missing link by demonstrating how Raman microscopy gives detailed information about chemistry and structure of the native cuticle on the microscale. We introduce an optimized experimental workflow, covering the whole process of sample preparation, Raman imaging experiment, data analysis, and interpretation and show the versatility of the approach on cuticles of a spruce needle, a tomato peel, and an Arabidopsis stem. We include laser polarization experiments to deduce the orientation of molecules and multivariate data analysis to separate cuticle layers and verify their molecular composition. Based on the three investigated cuticles, we discuss the chemical and structural diversity and validate our findings by comparing models based on our spectroscopic data with the current view of the cuticle. We amend the model by adding the distribution of cinnamic acids and flavonoids within the cuticle layers and their transition to the epidermal layer. Raman imaging proves as a non-destructive and fast approach to assess the chemical and structural variability in space and time. It might become a valuable tool to tackle knowledge gaps in plant cuticle research.

## Introduction

The cuticle is the outermost layer of the plant in direct contact with the environment. It consists of an epicuticular wax layer on top of a lipidized region of the epidermal cell wall. The various functions include controlling water and gas exchange, defense against pathogens, separating plant organs, and light protection and manipulation for the underlying tissues (Yeats and Rose, 2013; Martin and Rose, 2014; Pfündel et al., 2018). To fulfill these different tasks, lipidic and aromatic components build layers upon a carbohydrate-rich epidermal layer.

On top, the epidermal waxes are in direct contact with the atmosphere and maintain a clean surface to avoid reduced transmission of active photosynthetic radiation. Additionally, they scatter excess light (Pfündel et al., 2018) and impede the attachment of fungal spores and insect tarsae (Berto et al., 1999; Eigenbrode and Jetter, 2002). The beneath cuticular layer’s primary role seems to be controlling water diffusion (Martin and Rose, 2014). Depending on species and developmental stages, aromatic components impregnate the cuticle and transition to the epidermal layer (Sasani et al., 2021). In the last decade, the view of the cuticle shifted from a stand-alone layer on top of the epidermis to more integrated concepts (Yeats and Rose, 2013; Guzman et al., 2014b; Fernandez et al., 2016; Sasani et al., 2021). However, the chemical composition in context with the biological architecture of the plant cuticle is still under debate. To close this knowledge gap, we present Raman microspectroscopy as a valuable tool for cuticle research.

### Chemical Imaging: The Missing Link?

In the past, cuticles have been studied mainly through electron microscopy for elucidating their structure (Jeffree and Sandford, 1982; Wattendorff and Holloway, 1982; Tenberge, 1992; Lopez-Casado et al., 2007; Guzman et al., 2014a,c; Kwiatkowska et al., 2014; Segado et al., 2016), while the chemistry was revealed by lipid extraction and separation procedures (Franich et al., 1978; Hunt and Baker, 1980; Tulloch and Bergter, 1981; Jetter and Riederer, 1994, 2016; Kögel-Knabner et al., 1994; Prügel and Lognay, 1996; Oros et al., 1999; Villena et al., 1999; Jetter et al., 2000; Goodwin et al., 2003; Solovchenko and Merzlyak, 2003; Wen et al., 2006; Buschhaus et al., 2007, 2015; Szafranek et al., 2008; Guzman et al., 2014b; Guzman-Delgado et al., 2016; Bourgault et al., 2020; Moreira et al., 2020). However, both approaches faced the same problem: destroying the native structure by peeling/scratching off cuticles. Electron microscopy better retains the structure but linking it to chemistry is problematic as stainings can be affected by different infiltration into the sample (Fernandez et al., 2016). Extraction and separation yield detailed chemical information, but it can hardly be linked to the cuticle structure destroyed in the process. The heterogeneous nature of the cuticle, comprising compounds with different solubilities and high spatial variability, calls for approaches, which link chemistry with structure (Guzman et al., 2014b; Fernandez et al., 2016; Guzman-Delgado et al., 2016). Here, we show how Raman microscopy offers an *in situ* method that links the chemical to structural information on the mico-scale. It is already successfully used in plant sciences for studying flower (Gamsjaeger et al., 2011), stem (Gierlinger et al., 2008; Zeise et al., 2018), leaves (Baranska et al., 2006; Richter et al., 2011), fruits (Cai et al., 2010; Szymanska-Chargot et al., 2016), and roots (Schreiber et al., 2010; Heiner et al., 2018), but so far only a few Raman studies on cuticles are available (Littlejohn et al., 2015; Prats Mateu et al., 2016; Philippe et al., 2020; Sasani et al., 2021).

In this method article, we explore the potential of Raman imaging on cuticles of three plant species and provide an informative guide for successful applications. We share our experiences from sample preparation to spectra acquisition, from spectra analysis to image generation, and give new insights into the spectral interpretation of cuticle components.

## Materials and Methods

### Samples and Microtomes

**FIGURE 1:**
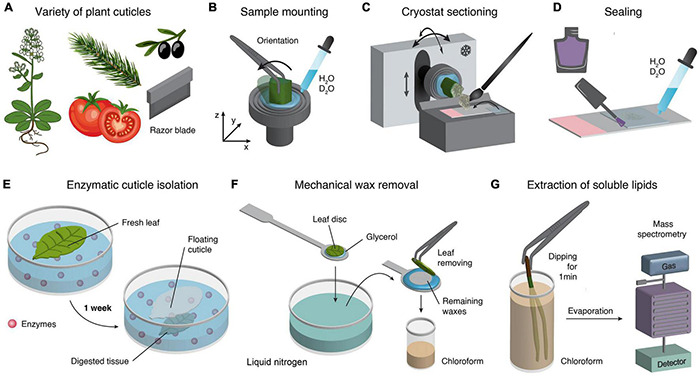
Cuticle sample preparation techniques. **(A)** For a wide variety of plant cuticles different sample preparation techniques are available. In a first step a sharp razor blade separates fast and easy areas of interest in form of small pieces for microtome sectioning. **(B)** For cryo-cutting, the mounting of a sample can be easily achieved by freezing a tissue on the sample holder. The advantage is that the sample orientation can be adjusted, and no embedding medium is needed. **(C)** Cutting in the cryostat allows the adjusting of the temperature of the chamber and the sample holder separately. Furthermore, the orientation of the sample holder can also be adjusted accordingly. **(D)** Cross-sections embedded on glass slides with a drop of water and coverslip and finally sealed with nail polish. **(E)** Enzymatic separation of cuticles (Vráblová et al., 2020). **(F)** Mechanical wax removal (Jetter et al., 2000). **(G)** Easy and fast extraction of soluble lipids by dipping samples into chloroform (Guzmán-Delgado et al., 2017).

Fresh samples (Figure 1A) and proper storage are essential to exclude unwanted chemical or biological reactions (oxidation, microbial growth, etc.). Before micro sectioning, cutting small blocks with a sharp razor blade is simple and straightforward (Figure 1A). To stabilize soft tissues, samples often need to be embedded in polymers or resins before cutting. However, the Raman signal of embedding media can superimpose with those from the tissue (Coste et al., 2021). Therefore, water-soluble polyethylene glycol (PEG) is recommended (Gierlinger et al., 2012; Prats Mateu et al., 2016). However, cryo-microtomes cut native samples in the frozen state without any treatment. Therefore we consider cryomicrotomy as the best sample preparation for Raman imaging and use a cryo-microtome (CM 3050 S) from Leica (Biosystems Nussloch GmbH, Germany).

### Cryosectioning and Sample Preparation

For successful imaging and reproducibility, selection of target regions (developmental and tissue-specific) and the orientation is crucial (Gierlinger et al., 2012; Butler et al., 2016). Thus, before mounting, it should be considered whether the abaxial or adaxial cuticle or the tip or middle portion of a needle or leaf is the area of interest. Furthermore, different sample orientations allow probing orientation of molecules with respect to stem axis or leaf surface. Here, the cryo approach provides easy adjustment of the sample’s angle during mounting or by moving the sample holder in the cryostat (Figures 1B,C).

Disposable blades are valuable and easy to handle; for example, the N35 blade (35°) from Feather (Japan) is particularly suitable for frozen tissues and allows the sectioning of flat, thin (1–20 μm) and relatively large sections. Using a trimmed, clean, and fine brush allows collecting the micro section after cutting and transferring onto a cooled glass slide. Next, the micro section is directly covered with a cooled coverslip in the cryostat (Figure 1C). Finally, the sample can be taken out of the cryostat and slowly accommodated to room temperature. A water drop might also be added. After rinsing several times with water, the coverslip is sealed with nail polish to avoid dehydration (Figure 1D). More information about sample preparation can be found in Gierlinger et al. (2012), Butler et al. (2016), Prats-Mateu et al. (2020).

### Other Cuticle Preparation Techniques and Their Potential for Raman Experiments

Most studies used sample preparation techniques other than cryo-sectioning, relying on different chemical and mechanical treatments. Recently (Vráblová et al., 2020) reported a modified enzymatic method, which isolates abaxial and adaxial cuticles and avoids the formation of wrinkles (Figure 1E). This method can potentially be applied to Raman studies because following the separation of the cuticle *in situ* would shed new light on the interface between the cuticle and the epidermal cell wall. In addition (Jetter et al., 2000) showed that a cryo-adhesive-based approach allows for mechanical isolation of the epicuticular wax film (Figure 1F). This technique can be employed directly under the Raman microscope equipped with a heating and freezing stage (e.g., Linkam, United Kingdom) to follow the separation *in situ*. Another study showed an easy and fast extraction method of soluble lipids from the cuticle surface by dipping the leaves directly in chloroform and subsequent analysis with mass spectrometry (Guzman-Delgado et al., 2016; Figure 1G). A very recent study by Chang and Keller (2021) used chloroform and hydrogen peroxide treatments to test the mechanical influence of the cuticle and skin cell walls during berry splitting. Lim et al. (2020) used cuticle-defective mutants with increased transpiration and reduced water potential to show the movement of salicylic acid toward the cuticle. They also used chloroform to extract the cuticular waxes. Here, Raman experiments before and after extraction would add tissue-specific and spatial information about the extraction progress and mutants.

### Raman Microspectroscopy

Raman spectroscopy probes molecular vibrations and changes in the polarizability of molecules (inducing a dipole) by using a monochromatic laser (Colthup et al., 1990). The excitation wavelength and the numerical aperture (NA) of the objective determine the spatial resolution. For example, using an oil objective (NA = 1.4) and a standard 532 nm or 785 nm laser, the theoretical resolution is about 232 and 342 nm, respectively. In addition, a short wavelength (e.g., 532 nm) is preferred because of the higher Raman intensity gained. However, if samples absorb at these wavelengths, fluorescence can swamp the Raman signal to levels where a high background masks all peaks. For example, in previous studies (Sasani et al., 2021), flavonoids often caused high fluorescence at 532 nm and scanning with 785 nm laser gave better results. Choosing the right laser wavelength is based on individual samples and trial and error.

#### Integration Time and Laser Power

If a sample cannot sustain laser power for the length of a measurement, then laser power, point density and integration time can be adjusted. A choice has to be made between the quality of the spectra and the spatial resolution of the image. For higher spectral quality, increasing the distance between measurement points reduces the time the sample is exposed to the laser. If the spatial resolution should not be compromised, then the laser power or the time the laser remains on a single measurement spot (called integration time) can be reduced. However, lower integration time means noisier spectra. In our experiments, laser powers of 10–30 mW and integration times of 0.01 seconds have proven optimal parameters for a wide range of samples. Time series measurements (0.01 s resolution) are suitable to check whether a sample is prone to laser degradation.

#### Avoiding Fluorescence

The most problematic drawback of Raman spectroscopy is fluorescence. This means that a molecule absorbs radiation at a particular wavelength, lifting electrons into a higher energy state. After falling back to the ground state, the electrons release energy as radiation and create more photons than Raman scattering. The consequence is that if the absorption of a sample coincides with the laser wavelength, fluorescence can mask the Raman spectrum (Colthup et al., 1990; Smith and Dent, 2005; Nebu and Sony, 2017). In this case, changing the laser wavelength, adding freshwater, or extracting some compounds from the sample can be helpful. If cells with chlorophyll are within the region of interest, a preliminary fast scan with short integration time is recommended to bleach the chlorophyll; the subsequent scan will be without fluorescence.

#### Polarizability and Molecule Orientation

One characteristic of Raman spectroscopy is that molecules with extended delocalized π-electrons (carotenes, cinnamic acids, flavonoids, stilbenes) induce bands with a very high Raman intensity (Schmid and Brosa, 1971; Schmid and Topsom, 1981; Felhofer et al., 2018; Maia et al., 2021). Therefore, even low quantities of molecules can be tracked within a sample and particularly in the cuticle. However, their signal can be so strong that the discrimination of other compounds is difficult, but changing the wavelength might be helpful (Bock and Gierlinger, 2019). Furthermore, a change of bond polarizability concerning laser polarization can be seen in crystalline structures like the wax layers. The highest polarizability is often along bond axes and allows statements about molecular order and orientation by Raman spectroscopy (Gierlinger et al., 2010; Sasani et al., 2021).

#### Water as a Native Embedding Medium

The substantial benefit of Raman microspectroscopy is the possibility of studying plants in their native (wet) state. The reason is that the water Raman intensity is relatively low and shows only one band. If an embedding medium other than water is used, its Raman spectrum can be subtracted in a post-processing step. In practice, however, the impregnation of embedding media varies with different tissues (Coste et al., 2021), and their spectral contribution cannot entirely be removed, causing spectral artifacts to remain. Therefore, it is advisable to use water or deuterium oxide as an embedding medium because of its relatively weak Raman signal and only one notable band at around 3400 cm^–1^. In addition, tracking the water distribution within different tissues and layers is possible (Horvath et al., 2012; Prats Mateu et al., 2016).

### Raman Imaging

A piezo motorized scan stage allows fast stitching of the entire sample, which supports selecting the region of interest for area scans (x,y). In addition, depth scans (z) are possible to elucidate the three-dimensional chemical distribution. However, in practice, quantitative depth scanning is only possible in more or less transparent samples, like silica protrusion in horsetail (Gierlinger et al., 2008). Before measuring, it is crucial to adjust and fix the sample on the scan stage according to the laser polarization. Rastering a sample with a laser point by point creates a hyperspectral data cube, including the spatial position (x,y,z) and the molecular fingerprint (spectrum). Finally, the hyperspectral data cube allows the calculation of Raman images based on univariate and multivariate approaches (Gierlinger, 2018).

#### Image Processing

Applying different image processing approaches can reveal the multicomponent chemical structure. Before image processing, it is necessary to remove cosmic rays by a software algorithm and check whether intensity differences are due to chemical differences or the focal plane (sample flatness). If the measured surface has not been even, regions out of focus can appear with less signal and cannot be separated from areas with a lower number of molecules. As the multicomponent spectra with overlapping bands are often difficult to explore by band integration and marker bands (see section “Results and Discussion” Step1), it is helpful to include multivariate methods taking care of the whole wavenumber range instead of selected wavenumbers/bands. However, before applying these methods, a suitable background correction algorithm (see e.g., Prats-Mateu et al., 2020), which works on every hyperspectral data cube pixel, is recommended.

#### True Component Analysis and Mixture Analysis

The “True Component” post-processing function included in the WITec5 software establishes the number of components in a dataset, locates them in the image, and differentiates their spectra. The function calculates the hyperspectral dataset as a linear combination of the most different spectra with a basis analysis algorithm (Dieing and Ibach, 2011). The number of components was increased step by step as long as different and meaningful spectra could be retrieved. To further analyze the multicomponent nature of the derived spectrum (layer), we modeled in the next step the “layer spectra” as a linear combination of measured reference spectra using the Orthogonal Matching Pursuit (Pati et al., 1993). This algorithm compares the spectral signature of the distinguished cuticle layers with spectra of known compounds of a reference library, including 326 entities, from aromatics to lipids to carbohydrates. The orthogonal matching pursuit (OMP) is an iterative approach, running fast enough to handle hyperspectral datacubes. A member from the spectral reference library that best correlates with the residual of the linear combination of references selected in the previous iterations is sought at each iteration. This reference is then added to the predictor set. A termination criterion such as a threshold for the residual error or a maximum number of iterations stops the search for additional members. The predictor set of the final model contains the selected members of the mixture, and the coefficients indicate the frequency of the respective compounds. Completeness and scaling of the spectral library, the analyzed spectral range, preprocessing (most important baseline correction), among others, have a substantial effect on the result and must be carefully evaluated. Yet, coupled with an exhaustive assessment of the plausibility of the result and testing the robustness of the fit, mixture analysis is an invaluable tool in analyzing complex biological spectra, like the cuticle.

## Results and Discussion

We use Raman images of cuticles of spruce, tomato, and Arabidopsis to show the procedure of image acquisition and analysis as well as cuticle spectral variability across plant species, organs, and layers. We offer an extensive analysis of the cuticle of spruce needles with our optimized experimental workflow, including:

1. Initial univariate exploration by band integration.
2. Determination of molecular orientation.
3. Multivariate image decomposition.
4. Mixture analysis of the resulting spectra.

We then discuss special issues of fluorescence and aromatic signal dominance on cuticles of tomato and show the cuticle of Arabidopsis as a counterexample with much fewer phenolic compounds.

### Step 1–Exploring the Sample via Band Integration

**FIGURE 2:**
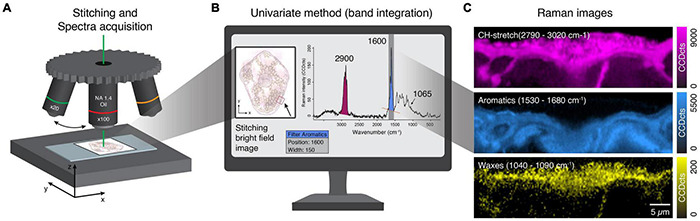
Stitching, Raman spectra acquisition, and image generation. **(A)** Different objective lenses for stitching and high-resolution Raman imaging. **(B)** Stitched bright-field image based on the x20 objective lens provides a good sample overview to select and show regions of interest. Univariate spectra analysis uses filters to plot Raman intensity of selected bands (wavenumber ranges). **(C)** Calculated Raman images based on Raman intensity of different wavenumber ranges corresponding to all components (CH stretching) and selected chemical groups (aromatics, waxes). See also Raman TV (Supplementary Video 1).

A thin section of a needle of spruce was imaged with a Raman microscope (Figure 2A). The resulting Raman image displays the CCD counts of the Raman band integrated over a defined wavenumber range at every pixel (Figures 2B,C). Pixels with higher band intensity are usually highlighted in brighter colors than areas with low signal. Since the bands of biological samples are often unknown beforehand, the Raman image is explored by changing the center wavenumber, that is, moving the slider through the spectrum. Then, the Raman image constantly changes from noise to clear pictures, similar to tuning a TV to a specific program (Supplementary Video 1). Peaks of biological samples often have bandwidths of 20–30 cm^–1^ (FWHM), which is a reasonable first choice for the slider width. This way, the sample is explored, and areas of similar chemistry can be found. They may correspond to structures seen in the visual image but can also reveal hidden structures not visible in light microscopy.

We used the strong CH-stretching band centered at 2900 cm^–1^ to visualize all organic components throughout the whole tissue (Figures 2B,C). As this band’s intensity also depends on the focus of the image, it can be used to check the quality of the scan before further analysis. We also detected a solid aromatic signal centered at 1600 cm^–1^, clearly visible in the inner part, as it is also the predominant spectral feature of lignin. In addition, we used a band representing the wax layer in the outer part of the tissue centered at 1065 cm^–1^ (Figures 2B,C). The strongest bands give clear pictures but often comprise contributions from more than one component and are suitable for visualization, but often not so informative. Weaker bands (e.g., 1065 cm^–1^) usually result in noisy images but can have high selectivity if they represent a marker band for a specific component.

### Step 2–Determining Molecular Orientation Within the Sample by Laser Polarization Experiments

By using a polarized monochromatic light source, the crystallinity and orientation of molecules can be probed. Controlling the orientation of the laser polarization relative to the sample orientation can provide spectra with information on the preferred orientation of molecules within the sample. This builds on the fact that the polarizability is normally greatest along a chemical bond. Raman polarization measurements have already been shown on biological materials (Cao et al., 2006; Gierlinger et al., 2010; Dong et al., 2021) and recently on cuticles of spruce needles (Sasani et al., 2021).

**FIGURE 3:**
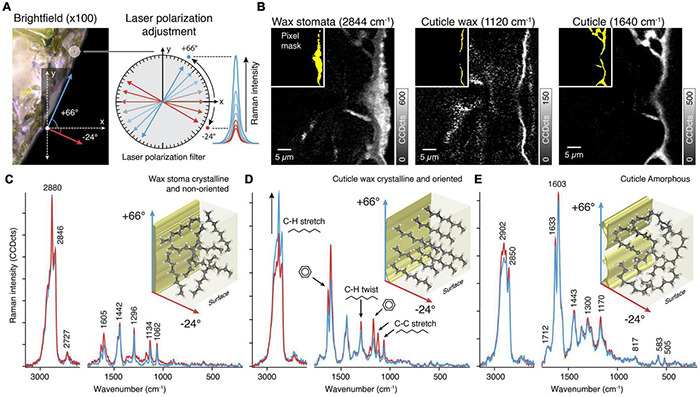
Orientation of molecules in plant tissues. **(A)** Changing the laser polarization allows probing molecular orientations by following the change in Raman intensity with respect to different laser polarizations angles. In the tilted investigated area the maximum and minimum intensity was found at +66° and −24°, respectively. As these angles represents the tilt of the sample, the molecules are orientated parallel and/or perpendicular to the surface. **(B)** The Raman images are based on integrating specific bands sensitive to laser orientation. The inlays (yellow) show pixel masks for extracting average spectra for the different layers. **(C)** Average Raman spectra of the wax in the stomata show sharp bands of a crystalline structure but no change in Raman intensity and thus no orientation. **(D)** The thin cuticle wax layer spectra show high crystallinity and oriented structures by changes in the Raman intensity of specific bands (C-H stretch, black arrow, C-H twist, and C-C stretch). **(E)** The underlying cuticle spectra are identical at different laser polarization and thus indicate amorphous components.

The first step is checking for molecular orientation in the sample. This is done by recording several Raman spectra of the same position but with varying polarization angles (e.g., 30° steps) and looking for differences between the spectra (Figure 3A). The subsequent acquisition of spectra exposes the sample over seconds to a focused laser beam. This can alter its chemistry, thus rendering the polarization experiment useless, because band intensity shifts cannot be solely related to orientation anymore. Hence it can be impossible to perform polarization experiments on samples prone to laser degradation and sample behavior have to be checked prior to such experiments (see Prats-Mateu et al., 2018 for details). In the shown example, peak maxima and minima were determined at the angles of +66° and –24°, which are related to the sample’s parallel and perpendicular orientation in relation to the surface. The spectral changes are consistent with previous measurements on cuticles and on pure references (Sasani et al., 2021).

The next step is to perform Raman imaging with the predetermined angles. We show average spectra extracted from the wax filling the stomatal antechamber, the epicuticular wax as well as the cuticle (Figure 3B). Two measurements were sufficient in our case, because we only observed spectral changes in the epicuticular wax layer. In case there are regions differing from each other in terms of peak maxima angles (that is their molecular orientation differs), each region has to be measured separately with its own angles.

Spectra of the stomatal wax (Figure 3C) showed no differences with regard to laser polarization but exhibited clear signs of wax crystallinity (sharp bands at 1134 and 1062 cm^–1^). This matches with prior observations that the plug consists of intermeshed and randomly oriented wax tubes (Jeffree et al., 1971; Jeffree, 2006).

On the contrary, spectra of the epicuticular wax showed differences (Figure 3D). Signal of CH stretches and C-C stretches showed opposite behavior, indicating that there is a net orientation of aliphatic chains. As the aromatic signal shows a concomitant change we conclude that coumaric acids are oriented along the wax chains. Both are oriented perpendicularly to the surface, corroborating previous ideas of wax orientation (Ensikat et al., 2006).

In the cuticle spectral differences between polarizations and the crystal bands are missing (Figure 3E). We therefore conclude on non-oriented and amorphous aliphatic chains in the cuticle.

This example shows that polarization experiments require more effort, may not work on some samples, but generate great insights into the molecular organization of the cuticle.

### Step 3–Revealing Hidden Layers and Components by Multivariate Analysis

Although simple band integration combined with polarization measurements already provides insights into the chemical structure of the cuticle, the spectral components often cannot be unambiguously determined. In addition, the overlap of Raman bands of different components is difficult to estimate when marker bands coincide. Then multivariate statistical methods come into play as they do not only consider single peaks, but include the whole spectrum for analysis. As an example, out of the many different multivariate methods (Felten et al., 2015; Gierlinger, 2018; Prats-Mateu et al., 2018; Sasani et al., 2021), we chose to show the very simple and fast to apply “True Component Analysis” (TCA) implemented in the Project FIVE software.

**FIGURE 4:**
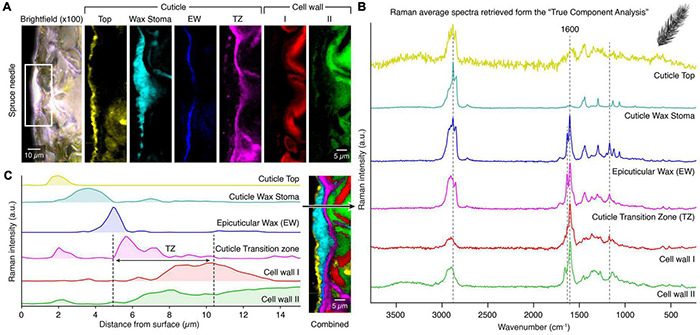
True component analysis of spruce needle cuticle **(A)** Bright-field image with the measured area marked with the white rectangle and the six gained Raman images by the “True Component Analysis.” First, four different components within the cuticle with varying chemical compositions. Second, two different cell wall layers. **(B)** Corresponding Raman spectra. **(C)** Raman intensity profile from the surface inwards to the epidermal cells. Note the special cuticle transition zone (TZ), which overlays with the epidermal cell wall. Legend: (EW) Epicuticular wax, (TZ) Transition zone.

On the spruce needle cuticle, this analysis revealed seven spectral components with different spatial distribution patterns based on various chemical entities of the cuticle (Figure 4A). The corresponding average spectra exhibit the chemical nature of these layers (Figure 4B). On top, a thin outer layer was identified with marked fluorescence and thus noisy spectra (Figures 4A,B, yellow). As a second layer, the wax sealing with distinct bands at 2900 and 1440 cm^–1^ was retrieved (Figures 4A,B, cyan). Also, the underlying epicuticular wax layer was separated with a thickness of only one micrometer (Figures 4A,B, blue). Below this, a lipid-rich layer, even extending to the inner walls of epidermal cells, was identified (Figures 4A,B, pink). The underlying epidermal cell walls show no lipid signal, but the analysis separated two individual components with different aromatic compositions (Figures 4A,B, red and green).

In Figure 4C, we show an additional feature of Raman imaging. The intensity of the different components can be displayed along a line from the outer surface inwards, representing the depth distribution in the cuticle. The different components display individual layers with minimal overlap and the transition zone (Figure 4C, black arrow). This novel method highlights the capability of Raman spectroscopy to determine the micro spatial distribution of chemical components inaccessible by many other methods.

### Step 4–Decoding the Spectra by Mixture Analysis

**FIGURE 5:**
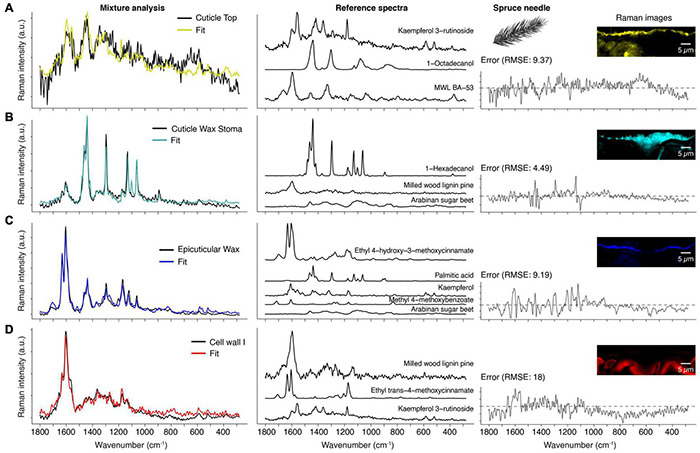
Mixture analysis of spruce needle spectra. Based on a reference library the spectra from the True Component Analysis are fit by a mixture alogorithm (see method part). **(A)** Cuticle top layer (black spectrum) compared with the fit result (yellow), which is a linear combination of a flavonol (Kaempferol 3-rutionside), saturated fatty alcohol (1-Octadecanol), and milled wood lignin (MWL BA-53). The residual error is shown on the right and the Raman images from Figure 4 for better orientation. **(B)** Spectrum of the cuticle wax sealing of the stoma (black) and the resulting fit (cyan) and reference spectra. **(C)** The thin epicuticular wax layer spectrum (black) and its fit (blue) and reference spectra. Note that this layer also has incorporated cinnamic acid and flavonol. **(D)** The first cell wall layer (red) is mainly fit by lignin, but also cinnamic acid and flavonol (see also Supplementary Figure 1).

Different chemical components are often co-localized in biological materials and can therefore not be separated with a simplistic analysis. Thus, the last step of our comprehensive approach was to identify the chemical identity of the received spectra by comparing them with reference compounds spectra from a library. For this purpose, we used a mixture analysis strategy based on the orthogonal matching pursuit, which models the experimental spectrum as a linear combination of selected compound spectra from a spectral reference library (see section “Methods” for details). In addition, we discuss the advantages of this method and point out some pitfalls and how to avoid them. The mixture analysis results shown in Figure 5 and Supplementary Figure 1 are based on the obtained spectra from the “True Component Analysis” of the spruce needle measurement (Figure 4).

#### The Fluorescing Top Layer

The first component is already an excellent example of the limits of this method because the experimental spectrum shows a high fluorescence background (Figure 5A). Baseline correction removes the elevated background, but this does not affect the high noise level. Consequently, the algorithm has little guidance for optimizing the fit. Despite this, the chosen references fit surprisingly well because the selected compounds make sense in the context of a spruce needle. For example, kaempferol-3-glycosides were found in the cell walls of spruce needles (Heilemann and Strack, 1990; Slimestad et al., 1992; Ganthaler et al., 2017). Although the spectrum alone cannot prove the occurrence of kaempferol at this position, the strong fluorescence of pure reference compounds (kaempferol, kaempferol-3-O-glycoside, and kaempferol-3-O-rutinoside) is an indication for the presence. Thus, researchers should not focus only on the fit and its error alone but also critically examine the reference spectra chosen by the algorithm.

Moreover, the exact compound identification in a complex mixture below the group level is challenging (e.g., group: flavonoids) because the algorithm takes the compound spectrum, which gives the best fit over the whole range, but the basic structure or origin is unclear. For example, flavones (e.g., kaempferol) can be distinguished from flavone glycosides (e.g., kaempferol-3-O-glycosides), but the glycoside nature cannot (e.g., it is impossible to differentiate between kaempferol-3-O-glucopyranoside and kaempferol-3-O-rutinoside). The second compound, octadecanol, represents the wax occurring at the top layer (Figure 5A). Finally, the fitted milled wood lignin spectrum is the third component, which underlines the aromatic nature of the unknown component(s).

#### Wax Sealing the Stomatal Entrance

Figure 5B shows the model result of the wax component, correctly identified as crystalline wax. The fitted compound 1-hexadecanol serves as a representative for waxes in general. Our library currently only contains C16 and C18 waxes; nevertheless, this is not problematic because the Raman spectra show very subtle changes concerning the number of carbons in the chain. The weak aromatic ring stretch at 1604 cm^–1^ is likely the cause for the inclusion of the milled wood lignin spectrum. However, cinnamic acid as the origin is more plausible due to the band at 1630 cm^–1^. Finally, arabinan is perceived as a component to account for the background in the experimental spectrum.

#### The Cuticular Layer

The fit in Figure 5C shows the method’s power because the cuticle’s experimental spectrum is fitted by five different compounds (Figure 5C). For example, the ferulic acid ester is the main component consistent with the literature, where p-cinnamic and ferulic acid are found in the cutin of fruits (Wang et al., 2016; Ding et al., 2020). Depending on the parameters, the algorithm often also chooses p-cinnamic acid esters (data not shown) because both acids have similar Raman spectra. The lipidic fraction of the cuticle is represented by palmitic acid (C16), in perfect agreement with the literature, which reports cutin being mainly composed of C16 and C18 fatty acids (Holloway, 1994; Lara et al., 2015). Kaempferol is singled out as representing a flavonoid component and is the dominant flavone in spruce needles (Ganthaler et al., 2017; Szwajkowska-Michałek et al., 2020). Also, several hydroxybenzoic acids were found in needles (Szwajkowska-Michałek et al., 2020), represented in our analysis by methyl-4-methoxybenzoate. Lastly, the algorithm also attributed a minor role to a polysaccharide. At least in cuticles of tomatoes, polysaccharides have been found (Lopez-Casado et al., 2007; Philippe et al., 2020).

#### The Spectrum of the Cell Wall

The cell wall spectrum is another prime example of capturing all main spectral contributors (Figure 5D). The method chose the milled wood lignin of pine as the best fitting lignin spectrum, which is very close to spruce, considering that hardwood lignin spectra are also in the library. The second component is a p-coumaric acid ester, representing coumarates and ferulates in the cell wall (clearly identified by intense bands at 1630 and 1180 cm^–1^). Again, a kaempferol glycoside was chosen, accounting for the bands at 1570, 595, and 521 cm^–1^.

### Tomato Cuticle as an Example for High Fluorescence

The previous section showed the spruce’s cuticle analysis, where reasonable Raman spectra could be obtained with 532 nm laser despite fluorescence. Nevertheless, tomatoes show a high tendency for fluorescence background, masking most of the Raman signal (see Supplementary Figure 2) and making the experiment prone to sample degradation and sample burning. Fluorescence occurs when the excitation wavelength is close to molecular absorption and can be avoided by changing the laser wavelength (e.g., 532 to 785 nm). Thus, we used a 785 nm laser and obtained spectra with much lower background and better signal-to-noise ratio.

**FIGURE 6:**
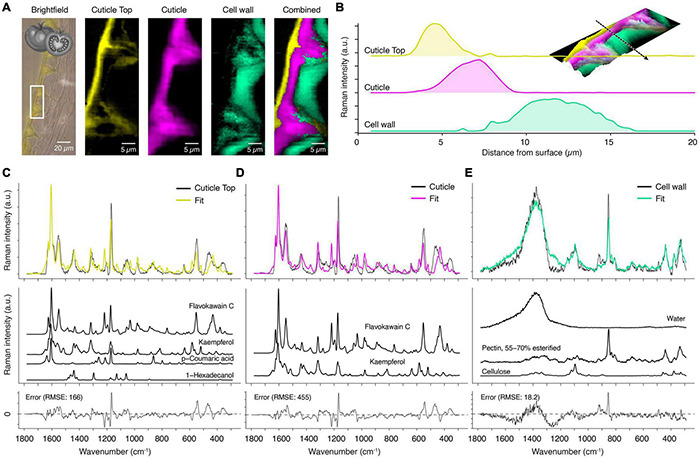
True component and mixture analysis of tomato cuticle. **(A)** Bright-field image with the measurement area (white rectangle), calculated component images and a combination of all. **(B)** Raman intensity profile along the dotted arrow of the various layers from the surface inwards (see inlay: 3D Raman intensity (in the z-direction) combined image). **(C)** For the fit of the cuticle top layer spectrum (yellow) a precursor of flavones, the chalcone flavokawain-C, an aromatic ketone, was chosen. The flavone kaempferol and p-coumaric acid are further aromatic compounds in the top layer. Finally, C-16 fatty alcohol (hexadecanol) was modeled into as the wax component of the top layer. **(D)** The main cuticle (pink) was composed of mainly chalcone and flavone. **(E)** The cell wall component (green) was explained as a combination of water, pectin and cellulose.

The “True Component Analysis” on the tomato measurement identified three main components (Figure 6A), from which the top layer and the underlying cuticle strongly overlap (Figure 6B). The spectra of these two look very similar, and most notably, the signal of waxes and fatty acids seems to be completely absent in the fingerprint region (Figures 6C,D). The reason is that some aromatic compounds have increased Raman cross-sections if they contain conjugated aromatic rings (Schmid and Brosa, 1971, 1972, 1973; Schmid et al., 1977; Schmid and Topsom, 1981; Schmid and Brodbek, 1983). The signal further rises if a charge transfer path coincides with one or more displacement coordinates (Zerbi et al., 1991; Del Zoppo et al., 1998; Tommasini et al., 1999). Therefore, almost all peaks in the cuticle spectra can belong to naringenin chalcone (Figures 6C,D, yellow and pink), the predominant flavonoid in tomato cuticles (Hunt and Baker, 1980; Luque et al., 1995).

Applying mixture analysis on the derived component spectra confirms that chalcones play the main role by fitting flavokawain C as the main component in the first place (Figures 6C,D, yellow and pink component). Naringenin chalcone was included in the library, but the algorithm chose the methylated variant (flavokawain C) and additional kaempferol. Inspection of the spectra shows that naringenin chalcone has a strong band at 1509 cm^–1^ which is absent in the experimental spectrum. The better fit of its methylated form is an indication that naringenin chalcone occurs in a bound state in the cuticle of the tomato, meaning that the molecule is covalently bonded to other chemical components over its OH-groups. However, the nature of these different components cannot be inferred from the Raman spectra and requires other methods. Yet, this shows that detailed insights into a molecular structure can be acquired by Raman spectroscopy.

In the top layer, the fit included beside the flavonoids also p-coumaric acid and 1-hexadecanol, a representative for the wax (Figure 6C, yellow spectrum). The third chemical component, the epidermal layer, is fitted with pectin as a major component. The fit suggests a high water content of the cell wall because the reference library also includes pure water spectra (Figure 6E). In this layer, polysaccharides become visible in the spectrum as the masking effect of the stong scatterers is absent [compare the cell wall spectrum of spruce, which had only aromatic components (Figure 5D)].

**FIGURE 7:**
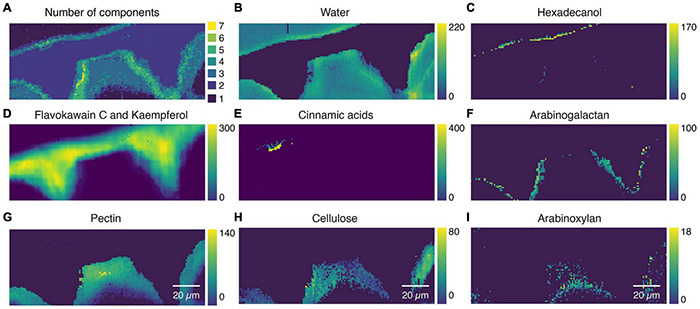
Mixture image analysis of tomato cuticle. **(A)** The number of components used from the library to fit the sample spectrum at every pixel. **(B)** Water distribution confirms the hydrophobic nature of the cuticle and reveals a hydrophilic interface toward the cell wall. **(C)** Epicuticular wax layer is imaged based on Hexadecanol distribution. **(D)** Flawokawain C and kaempferol are fitted as the two dominant spectral contributors within the main part of the cuticle. **(E)** Cinnamic acids accumulated toward the epicuticular wax layer. **(F)** Interface toward the epidermal cell wall included Arabinogalactan. **(G)** The epidermal cell wall was composed of pectin and **(H)** cellulose and **(I)** toward the lumen xyloglucan.

As the mixture analysis gave reasonable results with good fits on the extracted component spectra, we went a step further and applied the mixture analysis on the whole hyperspectral data set. The first interesting result is the number of references used to fit the spectrum at every pixel (Figure 7A). While the main cuticle was fitted using two components (flavokawain C, kaempferol), a transition layer (green, yellow) toward the epidermal and the top layer showed higher chemical heterogeneity. The water distribution image (Figure 7B) confirms the hydrophobic nature of the main part of the cuticle and reveals a hydrophilic interface toward the epidermal layer. The epicuticular wax layer was imaged by hexadecanol distribution (Figure 7C), the main part of the cuticle by flavokavain C and kaempferol (Figure 7D), while cinnamic acids seem to accumulate near the top layer (Figure 7E). In the interface toward the epidermal layer, arabinogalactan was chosen by the algorithm (Figure 7F), while the epidermal layer is represented by pectin (Figure 7G), cellulose (Figure 7H), and toward the lumen arabinoxylan (Figure 7I). A carbohydrate-rich cuticle layer toward the epidermis agrees with a current cuticle model (Heredia-Guerrero et al., 2014).

### The Components in the Shadow–Arabidopsis Without Strong Aromatic Scatterers

The previous examples, spruce (Figures 4, 5) and tomato (Figures 6, 7), included areas where spectra mainly showed a solid aromatic signal. Weaker Raman scatterers like linear aliphatics or polysaccharides contributed little to the spectra, although they are the primary mass component of the respective areas. Arabidopsis cuticles show less aromatic contributions, and so lipids and carbohydrates become more visible.

**FIGURE 8:**
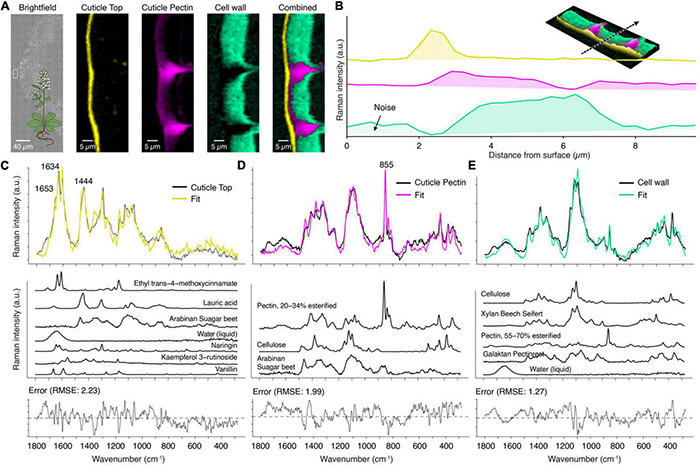
True component and mixture analysis of *Arabidopsis* cuticle. **(A)** Bright-field image with the measurement area (white rectangle), component images and a combination of all. **(B)** Raman intensity profile of the various layers from the surface inward (see inlay: 3D combined image along the dotted arrow). **(C)** The cuticle top layer showed a fitted spectrum (yellow) based on seven components, but still some misfits were observed. **(D)** The layer beneath was fitted based on pectin and polysaccharides. **(E)** The cell wall spectrum was modeled as a combination of cellulose, pectin, and hemicelluloses.

The “True Component Analysis” identifies three distinct areas: cuticle, pectin-rich layer, and the cell wall beneath (Figure 8A). The pectin-rich layer overlays with the top cuticle layer and the cell wall (Figure 8B). An aromatic signal centered around 1600 cm^–1^ is only visible in the spectrum averaged from the top layer (Figure 8C). Other studies also found that the cuticular waxes of Arabidopsis are chemically different from many other species because the main components are unsaturated diacids (Bonaventure et al., 2004; Yeats and Rose, 2013). This is in line with our recorded spectrum (Figure 8C), which has a distinct band at 1654 cm^–1^, originating from the C = C stretch of the unsaturated moieties. This band is absent in the wax spectra of spruce (Figure 5B, yellow) and tomato (Figure 6B, yellow).

The layer beneath shows only a weak signal of lipidic components (CH-stretching, not shown), and the mixture analysis does not fit in aliphatic components (Figure 8D). Instead, the assigned Raman band centered at 855 cm^–1^ reveals pectin as the main component (Figure 8D, pink) (Synytsya et al., 2003). This band is also visible in the spectrum of the cell wall, which shows a mixture of cellulose/hemicellulose and pectin (Figure 8E, cyan).

### Step 5–Band Shapes and Assignment of Major Cuticle Compounds

The bottleneck of many applications of Raman spectroscopy on biological materials is the interpretation of the spectra and assigning molecular structures to individual bands. This assignment strongly depends on the chemistry of the sample and can hardly be generalized. The desired result for most users is the assignment of *chemical components*. A vibrational band, however, first and foremost relates only to *a vibrational mode* (e.g., C = O stretch at 1720 cm^–1^), and such a mode can occur in many different molecules. Information about the sample’s chemistry is therefore required.

**FIGURE 9:**
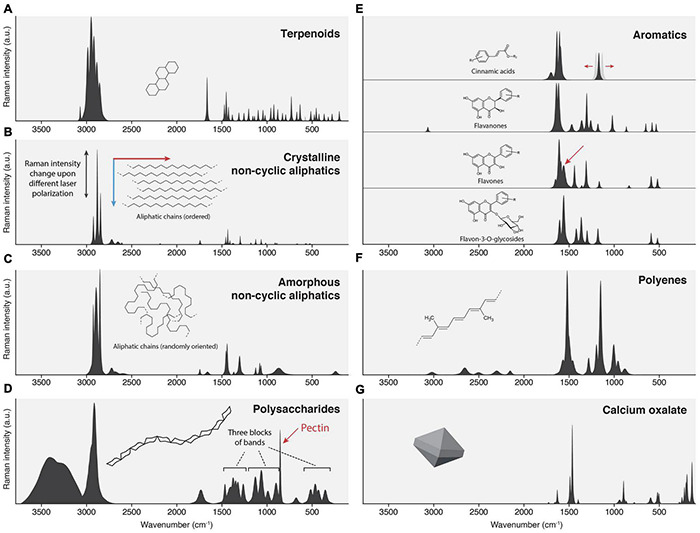
Idealized spectral patterns of several cuticular compounds. **(A)** Terpenoids: Strong CH stretch, many sharp bands in the fingerprint zone. **(B)** Crystalline waxes: Sharp bands, intense CH stretch, weak to medium CH bends, and C-C stretches. **(C)** Amorphous alkane chains in cutin: Similar wavenumbers like waxes, but broader bands; CH stretch is the strongest. **(D)** Polysaccharides: Notable OH stretch, undifferentiated CH stretch, fingerprint zone often containing three blocks of bands. **(E)** Aromatic compounds: Very weak CH stretches, dominant 1600 ring stretch. Most strong bands occur from 1660 – 1000 cm^−1^. **(F)** Polyenes: No CH stretches, three characteristic bands in the fingerprint zone (1520, 1150, and 1000 cm^−1^). **(G)** Calcium oxalate.

Many cuticle components are well characterized, and we discuss their spectral characteristics in the following paragraphs. Finally, we show idealized band shapes (Figure 9) and wavenumbers (Table 1) based on numerous measurements of pure compounds and compare them to experimentally observed values of cuticles.

**TABLE 1:** Idealized wavenumbers of standard cuticular components compared to experimental wavenumbers of cuticle spectra of spruce, tomato, and *Arabidopsis*. Assignments based on text.

Terpenoids	Waxes	Cutin	Carbohydrates	Cinnamic acids	Flavanones	Flavones	Flavon-3-O-glycosides	Polyenes	Cuticle 2 spruce Assignment		Wax tomato Assignment		Cuticle Arabidopsis Assignment	
			3400										3398	ν O-H (polysaccharides, water)

			3280										3247	ν O-H (polysaccharides, water)

								3200						

3070					3065				3063	ν C-H ring Φ2 (flavanones)			3066	ν C-H ring Φ2 (flavanones?)

2985														

2950														

			2940											

2920	2925	2925							2923	ν C-H (lipids)			2930	ν C-H (lipids, polysaccharides)

			2915										2914	ν C-H (lipids, polysaccharides)

		2895							2904	ν C-H (lipids)			2900	ν C-H (lipids, polysaccharides)

	2880												2882	ν C-H (lipids, polysaccharides)

2870														

2855		2850							2853	ν C-H (lipids)			2862	ν C-H (lipids, polysaccharides)

	2845													

	2720	2725							2725	δ C-H (lipids: overtone/combination)			2727	δ C-H (lipids: overtone/combination)

	2650							2650						

	2610													

								2500						

								2300						

								2150						

	1740	1745	1730											

				1700					1716	ν C = O (cinnamates)			1722	ν C = O (cinnamates, toward 1740 also ester groups of polysaccharides)

1660		1660											1654	ν C = C of unsaturated fatty acids

						1650								

				1635	1640				1633	ν C = C (cinnamic acids/esters)	1624	ν C = O (naringenin chalcone)	1633	ν C = C (cinnamic acids, esters)

					1615									

				1605		1610	1605		1603	ν C = C ring Φ8	1605	ν C = C ring Φ8a; ν C = C (naringenin chalcone)	1606	ν C = C ring Φ8

				1595		1590					1587	ν C = C ring Φ8b (cinnamic acids)		

								1570	1570	ν C = C ring Φ8 (cinnamic acids/flavones)				

						1560	1560				1552	ν C = C ring Φ8b (naringenin chalcone)	1560	ν C = C ring Φ8b + ν C = O (flavones)

								1520					1527	

1470					1470									

	1460							1460						

1450		1455	1455											

	1440	1440				1440	1420		1445	δ C-H (lipids), flavone mode	1440	δ C-H (lipids), flavone/chalcone mode	1441	δ CH (lipids)

1430														

	1410													

			1400											

	1380	1370	1375										1383	δ CH (polysaccharides)

					1360	1360	1365		1365	ν C-X ring Φ20a (flavones)				

multiple bands			1350											

			1320											

	1300	1305			1305	1310	1300		1303	γ_t_ CH (lipids); flavone mode	1310	γ_t_ CH (lipids); flavone/chalcone mode	1296	γ_t_ CH (lipids)

								1280						

strong band 800-500			1260		1260				1268	ν C-X ring Φ7a ferulic acids/ferulates	1261		1273	ν C-X ring Φ7a ferulic acids/ferulates

								1195			1205	ν_as_ C-C-O (coumaric acid/ester)	1204	ν_as_ C-C-O (coumaric acid/ester)

	1180				1180		1180							

				1170		1165			1171	δ CH ring Φ9a (coumaric acid/ester); Φ18a (flavone A-Ring); v C-C (ferulate)	1168	δ CH ring Φ9a (coumaric acid/ester, naringenin chalkone B-ring)	1170	δ CH ring Φ9a (coumaric acid/ester); Φ18a (flavone A-Ring); ν C-C (ferulate)

								1150					1131	ν C-C (lipids)

	1135	1125	1130						1122	ν C-C (lipids)			1114	ν C-C/C-O (polysaccharides)

		1080							1086	ν C-C (lipids)	1074	ν C-C (lipids)	1081	ν C-C/C-O (polysaccharides)

	1065	1065	1060						1063	ν C-C (lipids)	1053	ν C-C (lipids)	1063	ν C-C (lipids)

	1020				1020				1033	ν C-C (lipids)				

								1000					1007	

			990								981	flavone/chalkone mode		

								960						

	910		900										933	

	890												892	

								880			870	ν C-C + γ_r_ CH (lipids)		

		865			865									

			855										856	ν C-C/C-O (pectin)

						835			820	ν C-C (lipids)				

	670		670											

					650				648	δ C = C ring Φ6a (flavone A-Ring)	642	γ C = C ring Φ16b (naringenin chalcone A-ring)		

						590	590							

					580				583	ν C = C ring Φ1 (flavone A-Ring)				

											550	ν C = C ring Φ1 (naringenin chalcone A-Ring)		

					535									

			515			520	520		521	δ C = C ring Φ6b (flavone A-Ring)				

														

			470								459		443	

			430						413					

			355								357			

		240												

	205													

*Assignments based on text. ν: stretching; δ: in-plane bending; γ: out-of-plane bending; γ_t_: twisting; γ_r_: rocking; γ_w_: wagging; Φ: ring mode (Varsanyi designation, Varsanyi, 1969); C-X: ring mode involving substituents.*

#### Waxes

Epicuticular waxes consist of long-chain aliphatics (C20-C34), modified by acid-, aldehyde-, keto-, alcohol, and ester functional groups (Yeats and Rose, 2013; Lara et al., 2015; Chu et al., 2017; Zhu et al., 2018) and triterpenoids, the most common being α-amyrin, β-amyrin, ursolic and oleanolic acid (Haliñski et al., 2015; Lara et al., 2015; Chu et al., 2017). The most apparent feature of crystalline waxes is many sharp bands. This is due to the limited rotational freedom of the aligned chains or the rigid molecular body in the case of terpenoids. This alignment can often be seen in Raman spectra of different laser polarization (see also Figure 3D).

##### Terpenoids

The typical feature of terpenoid Raman spectra is very strong CH stretching bands (3000-2800 cm^–1^), the occurrence of bands around 1660 cm^–1^, and many sharp bands of more or less equal intensity in the fingerprint zone. The CH-stretching region is the strongest in the spectrum and consists of many sharp bands originating from various CH_2_ groups and lone hydrogens. Cuticle terpenoids can easily be distinguished from saturated alkyl chains by the signal from their C = C bonds, and the internal C = C stretch comes at ∼1660 cm^–1^, the vinyl C = C stretch at 1640 cm^–1^. Their hydrogens often produce a band near 3070 cm^–1^. The C = O stretch of acids is very weak in Raman and, therefore, not a useful band. The fingerprint region shows medium intense CH bending bands (∼1440 cm^–1^) and many needle-like bands of equal intensity, stemming from numerous CH and C-C modes of the stiff molecular skeleton (Figure 9A). Some modes can acquire substantial Raman intensity, and several strong bands are often observed in the region 800–500 cm^–1^.

##### Linear Alkyl Chains in the Crystalline State

Linear alkyl chains (cuticular waxes) can be identified by two sharp CH stretching bands and medium intense CH/C-C modes (2880, 2850, 1440, 1300, 1135, 1065 cm^–1^) (Figure 9B). There are fewer bands observed compared to terpenoids because the CH and C-C modes are chemically very similar. In crystalline alkyl chains, the CH-stretching region shows typical bands for CH_2_-stretching (antisymmetric: 2940, 2920 cm^–1^; symmetric: 2880, 2845 cm^–1^) (Colthup et al., 1990). Overtones of wagging and twisting modes cause a distinctive set of bands on the low-frequency side of the CH stretches (2720, 2650, 2610 cm^–1^). Carbonyl groups cause only weak Raman scattering (esters 1740 cm^–1^; aldehydes 1730 cm^–1^; ketones 1720 cm^–1^) and are often hidden under stronger aromatic carbonyls. A set of bands around 1440 cm^–1^ (CH bendings), one at 1300 cm^–1^ (CH twisting) (Colthup et al., 1990), and several C-C stretching modes in the range of 1150–800 cm^–1^ (Machado et al., 2012) are other characteristic bands of waxes in a cuticle. Below 800 cm^–1^, the spectrum is devoid of any notable bands. Varying the chain length does not affect the spectra much because the principal modes of the molecules are the same. Therefore, measurements of waxes with shorter chain lengths than usually found in most cuticles (C16 vs. C34) are valid. Because the chains are ordered in a parallel fashion in most crystalline forms, the intensity of CH and C-C bands differs relative to each other when probed by a linearly polarized laser. If the laser is parallel to the chain, the C-C stretching and CH-twisting will be stronger, while being orthogonal, the CH stretching band is by far the most intense.

#### Cutin Fatty Acids (Linear Alkyl Chains in the Amorphous State)

Cutin monomers are mainly C16 and C18 alkanoic acids (Holloway, 1994; Lara et al., 2015; Wang et al., 2016). Their spectra show the typical bands of alkylic chains, namely CH_2_ stretches (2926, 2851 cm^–1^), CH2 bendings and twistings (1455, 1440, 1303 cm^–1^). In contrast to cuticular waxes, they show broader bands because the amorphous structure allows many more conformations; therefore, the frequencies of individual modes vary stronger and blur into each other (Figure 9C). However, the CH_2_ and C-C modes are still at similar wavenumbers as in the crystalline state, so the principal distinction is made by spectral shape (see Figures 3D,E). Ester carbonyls are seen as weak bands at 1740 cm^–1^, the free acids at 1660 cm^–1^. The cuticle of Arabidopsis seems to be atypical (Yeats and Rose, 2013) and consists mainly of unsaturated diacids (Bonaventure et al., 2004). They show a strong Raman band at ∼1660 cm^–1^, stemming from the C = C stretch (see also Figure 8C).

#### Polysaccharides

Polysaccharides may function as the scaffold for lipids (Guzman et al., 2014b). Pectin, hemicelluloses (e.g., xyloglucan), and cellulose are reported in cuticles (Lopez-Casado et al., 2007; Guzman et al., 2014a; Philippe et al., 2020). Although plant polysaccharides are diverse compounds, their Raman spectra do not differ that much because the involved chemical bonds are essentially the same (OH, CH, C-C, C-O). Figure 9D shows a typical Raman spectrum. The high number of OH-groups causes a Raman band of notable intensity (∼3400, 3280 cm^–1^). It is neighbored by a high CH-stretching peak, which is often not resolved into individual bands. The band position is around 2915 cm^–1^, well set apart from the lower-lying stretches of alkyl chains centered at 2880 cm^–1^. Esterification can result in a weak to medium Raman band at ∼1730 cm^–1^. The fingerprint area shows three separated blocks of bands: CH bending modes (1500–1250 cm^–1^), C-O/C-C stretching modes (1180–900 cm^–1^), and various bending and twisting modes (500–350 cm^–1^). Pectin has a distinct Raman band at 855 cm^–1^ (Synytsya et al., 2003).

#### Aromatic Compounds

A variety of aromatic compounds is found in cuticles: cinnamic acids, hydroxybenzoic acids, benzyl esters, phenethyl esters, phthalate esters, and several flavonoids are reported (Kisser-Priesack et al., 1990; Luque et al., 1995; Laguna et al., 1999; Steinbauer et al., 2009; Bernal et al., 2013; Zhu et al., 2018). Aromatic rings are typically identified in Raman by a strong band at 1600 cm^–1^. If the ring is part of a delocalized π-electron system, the Raman cross-section of the whole conjugated system is significantly increased (Schmid and Brosa, 1971, 1972, 1973; Schmid et al., 1977; Schmid and Topsom, 1981). Cinnamic acids and flavonoids contain extended π-systems and correspondingly cause intense Raman peaks. It is important to note that all modes having bond movement of the charge-transfer-path in charge-transfer systems are enhanced (Tommasini et al., 1999). The result is that even small quantities of cinnamic acids and flavonoids cause strong Raman bands and, at some point, start to mask bands from non-enhanced molecular structures in a similar way as it is observed in lignin (Bock and Gierlinger, 2019). Furthermore, judging from absorption spectra, aromatic compounds are often responsible for fluorescence in Raman spectra if absorption bands hit the excitation wavelength or overtones (Bock, 2020). In the cuticle, aromatic compounds are primarily identified by bands in the region 1650–1550 cm^–1^. Many of them have no notable bands in the CH stretching region (Figure 9E).

Cinnamic acids show two strong bands at 1630 (C = C stretch) and 1605 cm^–1^ (ring stretch), esters an additional weak band at 1700 cm^–1^ (C = O stretch). Cinnamic acids show only a few other bands in the range 1500–1000 cm^–1^ and only weak to very weak bands below 1000 cm^–1^ (Figure 9E). Thus, the nature of the ring system can often be deduced from characteristic bands. However, in the case of cinnamic acids, ferulic acids/esters show an unusual strong band at 1180 cm^–1^, which is usually a marker band of coumaric acids/esters and is typical for para-substituted aromatic rings (Varsanyi, 1969). Since all other bands are so weak or hidden, these two cannot be separated with confidence (see also Figures 5C,D, where each of them is selected by mixture analysis).

Flavones and chalcones often have a very strong marker band at 1560 cm^–1^ (Figure 9E, arrow). They share a very strong band at 1600 cm^–1^ with all other conjugated aromatic compounds. In contrast to cinnamic acids, flavonoids show more strong bands over the whole fingerprint range; notable is a characteristic set around 600 cm^–1^, which is often stronger than modes of other molecules in this range, thus enabling confirmation of a flavone structure. Based on our observation, flavanones show less Raman intensity, and we attribute this to the reduced size of the conjugated system (the B-ring is not in conjugation). The consequence is that it might be more challenging to identify them in mixtures.

In contrast to flavones, weak CH stretches are often observed. We also note a difference in the spectra of flavones and their respective 3-O-glycosides. DFT-Studies show that the B-Ring is rotated out of the plane, reducing its conjugation with the chromone system (Cai et al., 2013; Paczkowska et al., 2015). Our interpretation is that this increases the relative intensity of A-ring modes in the spectrum and results in flavone-3-O-glycoside spectra being notably different from their respective aglycones (see Figure 9E).

#### Polyenes

Polyenes are identified by two characteristic bands, the C = C stretching (1520 cm^–1^) and C-C stretching (1150 cm^–1^) vibrations (Figure 9F). The 1000 cm^–1^ band is a more complex displacement, best described as CH_3_ wag (Novikov et al., 2021). Overtones and combinations can be seen at >2000 cm^–1^.

#### Calcium Oxalate Cystoliths

Calcium oxalate cystoliths were found in the lowest cuticle layers or directly underneath (Fink, 1991; Pennisi et al., 2001; Sasani et al., 2021), where one function is light scattering (Gal et al., 2012). The bands of calcium oxalate monohydrate are shown in Figure 9G. Mineral crystals, in general, have sharp bands stemming from rigid molecular structures and have strong bands at low wavenumbers (these are motions involving the heavy atoms like Ca).

#### Spectral Assignments of Three Experimental Spectra

In the last step, we compare the band assignments of pure, idealized components with spectra of the “True component analysis” as discussed in step 3 of this manuscript. Note that mixture analysis (step 4) and band assignment via pure reference compounds (step 5) are complementary. Mixture analysis results indicate certain compounds which should be checked for plausibility with known assignments. Conversely, individual band assignments should be cross-checked by modeling spectra of assumed substances into the experimental spectrum.

Table 1 shows a comparison of wavenumbers of chemical components with one spectrum of a cuticle from each of the three samples discussed so far. We chose a cuticle component of the spruce needle, the wax of the tomato, and the cuticle of Arabidopsis. We observe that our idealized wavenumbers, derived solely from pure reference compounds, match well with experimental cuticle spectra. We see this as a confirmation of our new approach using averaged and adjusted wavenumber sets to account for variability within a chemical group.

The assignments are now adjusted for the compounds known to occur in a cuticle. This means that chemical groups can be narrowed down. For example, in the tomato wax, the band at 1560 cm^–1^, indicating flavones and chalcones, can be narrowed down to naringenin chalcone or a very similar substance, with the help of mixture analysis. Hence the assignment specifically states “naringenin chalcone.”

The multicomponent nature of the needle cuticle of spruce (see Figure 5) is reflected in its band assignment. The lipidic fraction is best seen in the high wavenumber region (∼3000 cm^–1^), whereas most of the observed bands in the fingerprint region (∼1500–0 cm^–1^) are from aromatic compounds. Thus, the bands around 3000 cm^–1^ can be assigned to aliphatic components, in this case mainly cutin esters. However, a small band at 3063 cm^–1^ is of aromatic origin. Based on our observation that flavones, cinnamic and benzoic acids do not show bands in this region at 532 nm, we assign this band to flavanones, which display bands in this region and are still sufficiently strong scatterers to be seen. However, this does not exclude other aromatic compounds which might be undetermined yet. The strong band at 1603 cm^–1^ is unspecific for (conjugated) aromatic compounds. The bands 1633 and 1171 cm^–1^ are assigned to ferulic and or p-coumaric acid, while the band at 1268 cm^–1^ seems to be exclusively from ferulic acid. The presence of flavonoids is deduced from the shoulder at 1570 cm^–1^ together with bands at 648, 583, and 521 cm^–1^, which are assigned to various modes of the flavone A-ring. Lipids show only two clear bands, 1439 and 1303 cm^–1^, although both fall together with flavone modes, the bands being regarded as mixtures of both.

Compared to spruce, the wax of tomato shows a simple spectrum–simple because almost all bands are caused by a single chemical component, naringenin chalcone or a very similar structure (see also mixture analysis, Figure 6), in agreement with previous studies (Piringer and Heinze, 1954; Hunt and Baker, 1980; Luque et al., 1995). At 785 nm, the presence of lipids can only be derived from the bands at 1440, 1310, and 870 cm^–1^, although flavones/chalcones also have bands at these wavenumbers.

The assignment of the Arabidopsis cuticle is a little different because it also includes polysaccharides. These can be seen due to the weaker scattering of aromatic compounds in comparison to spruce or tomato. Polysaccharides and water cause the strong OH bands (3398, 3247 cm^–1^), the weak band at 3066 cm^–1^ is of aromatic origin, and the neighboring CH stretches come from polysaccharides and lipids. The bands at 1722, 1633, and 1606 cm^–1^ indicate cinnamic esters. There is an additional band at 1654 cm^–1^, which either comes from a C = C in conjugation with a ring (e.g., a cinnamyl alcohol moiety) or from an unsaturated fatty acid. We have not found studies reporting cinnamyl alcohols in the cuticle of Arabidopsis, but unsaturated cutin monomers were found by GC-MS (Bonaventure et al., 2004). We, therefore, assign this band to the C = C stretch of unsaturated fatty acids and do not count on the result of the mixture analysis in this case (Figure 8C). Polysaccharides are seen at 1380 cm^–1^, and pectin displays its marker band at 856 cm^–1^. We interpret the bands at 1131 and 1063 cm^–1^ as signs for crystalline aliphatic components (waxes). Furthermore, the measurement was performed with a laser polarization perpendicular to the surface, enhancing the C-C stretches if the chains are in line with the laser (see Figure 3 for wax orientation). This indicates that these waxes are also oriented in the cuticle or on top of it, although a more rigorous determination is required.

## Summary and Outlook

This method article highlights Raman microscopy as an *in situ* method to study the relationship of chemistry and structure on the microscale. We present a comprehensive workflow starting with the sample in hand and ending with detailed insights into chemical compounds and their spatial distributions within different cuticle layers. Native sample preparation, label-free imaging, detailed chemical information about all involved molecules and in context with the anatomical structures are essential assets of the Raman imaging approach. The examples confirm that even the molecule orientation can be deduced in an about 500 nm thick epicuticular wax layer (Figure 3), and transition and top layers can be distinguished with a spatial resolution of 300 nm and minor changes in composition (Figures 4–8). Furthermore, we show and discuss that especially aromatic components are strong Raman scatterers and can therefore be tracked even in small amounts. But in fact, researchers even pushed the limits of tracing compounds in attomolar concentrations (Yang et al., 2016) and sub-nanometer resolutions (Chen et al., 2019; Lee et al., 2019), showing the potential of the Raman toolbox.

We worked through cuticles of a spruce needle (Figures 3–5), a tomato peel (Figures 6 and 7), and an Arabidopsis stem (Figure 8), revealing common features as well as differences. The differences start already in the measurement setup, as cuticles with a higher amount of aromatic components (e.g., tomato) are more prone to sample degradation and more challenging to get spectra with a good signal-to-noise ratio using the 532 nm laser. So the 785 nm laser was the best choice for the tomato cuticle, although we lost spatial resolution. At high concentrations, aromatic components start to mask bands from non-enhanced molecular structures like carbohydrates and lipids. Therefore, probing different developmental stages of the cuticle might be interesting. Anticipating that lipids and carbohydrates are first deposited, the aromatic impregnation can be followed step by step. Or moving backward, using different solvents to see effects of the extraction procedure on the sample as well as characterizing the extracted components. Chemical environments, as well as bonding, can also be reflected in the Raman spectra. The here presented mixture analysis (orthogonal matching pursuit) of the native tomato cuticle spectrum suggested that naringenin chalcone is covalently bonded to other chemical components over its OH-groups as the methylated form better explained the spectrum (Figure 6). The power of this hyperspectral analysis was also proven on the hyperspectral dataset by spatially resolving even tiny layers with a high chemical precision (Figure 7). We showed the power of such methods with a library including the most relevant reference spectra in high quality but also displayed on the Arabidopsis cuticle that a thorough check of the results due to many possibilities and influence factors is necessary. As additional help for successful Raman spectra interpretation, we derived band shapes of the main cuticular component groups and their assignment (Figure 9 and Table 1).

**FIGURE 10:**
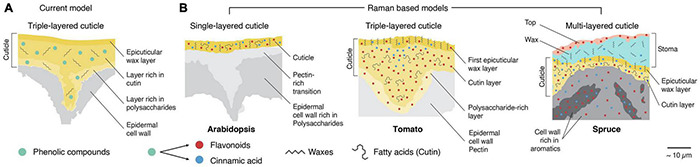
Extended models for different plant cuticles based on the Raman results. **(A)** The current model shows three different layers. An epicuticular wax layer is followed by a layer rich in cutin and a third rich in polysaccharides. Note that this model puts phenolic compounds mainly in the second layer. Model adapted from Heredia-Guerrero et al. 2014. **(B)** Models based on our Raman results (Figures 3–8). Left, *Arabidopsis*, showing only a single cuticle layer with additional flavonoids and cinnamic acids. Waxes are presumably crystalline and oriented. In the center, the triple-layered cuticle of tomato is shown. The top layer is composed of oriented waxes and cinnamic acids, while the subjacent layer is rich in amorphous cutin. Interestingly, cinnamic acids were mainly detected in the cuticle bulge and close to the top layer in this layer. The third layer is rich in polysaccharides. Flavonoids are found throughout the whole cuticle. On the right, the complex, multilayered model of a cuticle at a stomatal opening of the spruce needle is shown. A thin layer rich in flavonoids is observed on the top of the stoma wax (cyan). However, the stoma wax sealing seems to contain only a tiny amount of cinnamic acids and consists mainly of crystalline but random oriented wax crystals (see Figure 3C). In contrast, the underlying epicuticular wax layer (generally on the surface) shows clear signs of oriented crystalline waxes and co-oriented cinnamic acids (see also Figure 3D). The last cuticle layer is rich in amorphous fatty acids and aromatic compounds (flavones and cinnamic acids). Those aromatics transition into the subjacent cell walls where they co-localize with lignin but are not found in deeper-lying cells.

Based on the calculated Raman images, schematic models can be derived with unprecedented details and compared with current cuticle models (Figures 10A,B). Imaging micro sections allows to include the underlying epidermis and probe the transition or look at special cases like the cuticle above a stoma. What is seen at first sight is the diversity of the three investigated cuticles in dimension, layering, and chemistry. In Arabidopsis, we could only derive one layer with a relatively low amount of aromatic components (Figure 10B, Arabidopsis). Between the cuticle and cell wall, a pectin-rich layer was imaged. In the native tomato also a pectin-rich epidermal wall was found, but additionally a polysaccharide layer with flavonoids as a transition zone (Figure 10B, Arabidopsis). The high impregnation with chalcones was found throughout the whole tomato cuticle. These increased amounts probably mask other components present in lower entities, and cinnamic acids and lipids were only detected in the top region. Cinnamic acids accumulate near the epicuticular wax layer, and then transit into flavonoids; this is also a feature of the multilayered spruce cuticle (Figure 10B, spruce needle).

In contrast to the other examples, the cuticle in spruce needles is built upon a lignified epidermal cell wall, which is additionally impregnated with flavonoids found in the cuticle (Sasani et al., 2021). In spruce needles, the periclinal cell walls often present themselves as chemical hybrids of cell walls and cuticles, which we interpret as an additional reinforcement of the outermost cells. The complex cuticular setup of the spruce needle is even topped at the stomatal opening by two additional layers (Figure 10B, spruce).

Among the three derived Raman models, the tomato fits very well with the current model of the cuticle by distinguishing the epicuticular wax layer and a carbohydrate-rich transition layer towards the epidermis from the main part of the cuticle. But we now add detailed information about the aromatic components, which have so far only been indicated in the primary layer. Although our three investigated cuticles differed in their setup and layering, we can derive underlying chemical concepts: (1) cinnamic acids are co-localized with waxes and directly contact the outside world. (2) underlying layers are composed of amorphous cutin esters and contain flavonoids in addition to cinnamic acids. (3) these compounds stop at the border in unlignified cell walls (Arabidopsis, tomato) but gradually fade into the lignified cell walls.

With these measurement examples and informative guide we hope to spur the community to include Raman imaging in their cuticle research. Diving deeper into cuticle structure and revealing more hidden features will advance a comprehensive and holistic biological understanding of the plant cuticle.

## Data Availability Statement

The raw data supporting the conclusions of this article will be made available by the authors, without undue reservation.

## Author Contributions

NG, MF, and PB conceptualized the article and wrote the manuscript. NG and PB analyzed the data and interpreted the results. MF illustrated the figures. KM programmed the R-interface for the mixture analysis approach. All authors discussed the results, read and approved the article.

## Conflict of Interest

The authors declare that the research was conducted in the absence of any commercial or financial relationships that could be construed as a potential conflict of interest.

## Publisher’s Note

All claims expressed in this article are solely those of the authors and do not necessarily represent those of their affiliated organizations, or those of the publisher, the editors and the reviewers. Any product that may be evaluated in this article, or claim that may be made by its manufacturer, is not guaranteed or endorsed by the publisher.
