# Supplementary material for: A Guide to Elucidate the Hidden Multicomponent Layered Structure of Plant Cuticles by Raman Imaging
Source: Front Plant Sci. 2021 Dec 17;12:793330. doi: 10.3389/fpls.2021.793330 (PMC8718554; doi:10.3389/fpls.2021.793330)
Supplement: Supplementary file 2 [file Data_Sheet_1.PDF]

Supplementary Information for

A guide to elucidate the hidden multicomponent layered structure of plant cuticles by Raman imaging

Peter Bock[1], Martin Felhofer[1], Konrad Mayer[1], and Notburga Gierlinger[1*]
[1]Institute of Biophysics, Department of Nanobiotechnology, University of Natural Resources and Life Sciences, Vienna, Austria

Additional spectra of the mixture analysis of the spruce needle

Figure S1 A shows the fitted spectrum (pink) of the transition zone between the cuticle and epidermal cells. The original spectrum (black) is an average of two spectra retrieved from the "True Component Analysis" because the analysis found two areas but very similar for this zone. Therefore, we combined both images and averaged the spectra. The fitted spectrum (pink) consists mainly of cinematic acid, flavone, and coniferyl alcohol benzodioxan. Besides the cell wall layer spectrum, shown in Figure 5D, the second layer (see Figure 4A and B) shows other phenolic compounds (Figure S1 B).

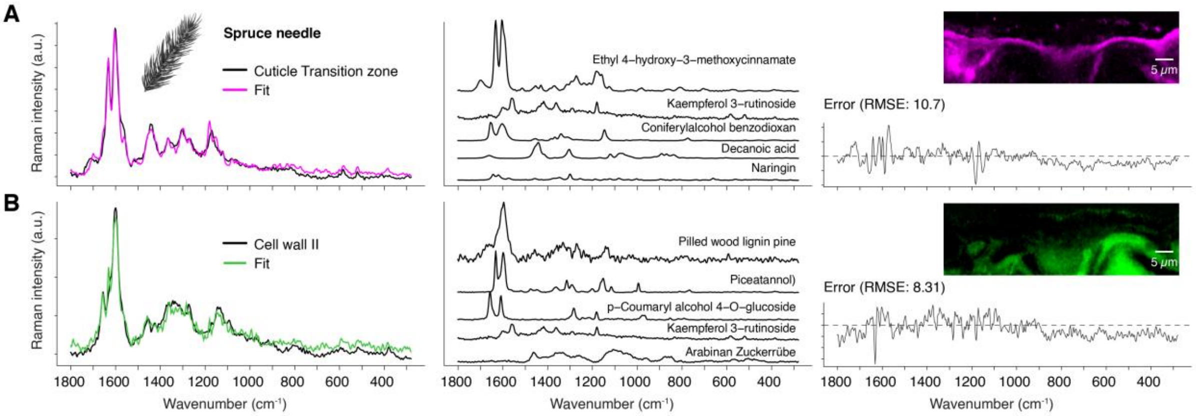

Figure S1. Mixture analysis of the spruce needle measurement. The black spectra are from the "True Component Analysis" used for the mixture analysis (see method part). (A) Cuticle transition zone (black spectrum) compared with the fitted spectrum, which is a composition of cinnamic acid and flavonol. The residual error is shown on the right and the Raman images from Figure 4 for better orientation. (B) The fit of the second cell wall layer shown in Figure 4A and B.

Tomato measurement with a 532 nm laser

Figure S2 shows the results of the measurement of the tomato cuticle with a 532 nm laser. The first image shows the sensitivity of the compounds to the 532 nm laser radiation because the distinctive squares correspond to the rastering of the sample. This means a chemical change and degradation occurred during the measurement, which is also reflected in the other compounds, resulting in noisy images. In addition, the laser power during this measurement was set to 20 mW and the integration time was 0.1 seconds per pixel. In the future, decreasing the laser power and integration time can help to obtain better spectra with a 532 nm laser.

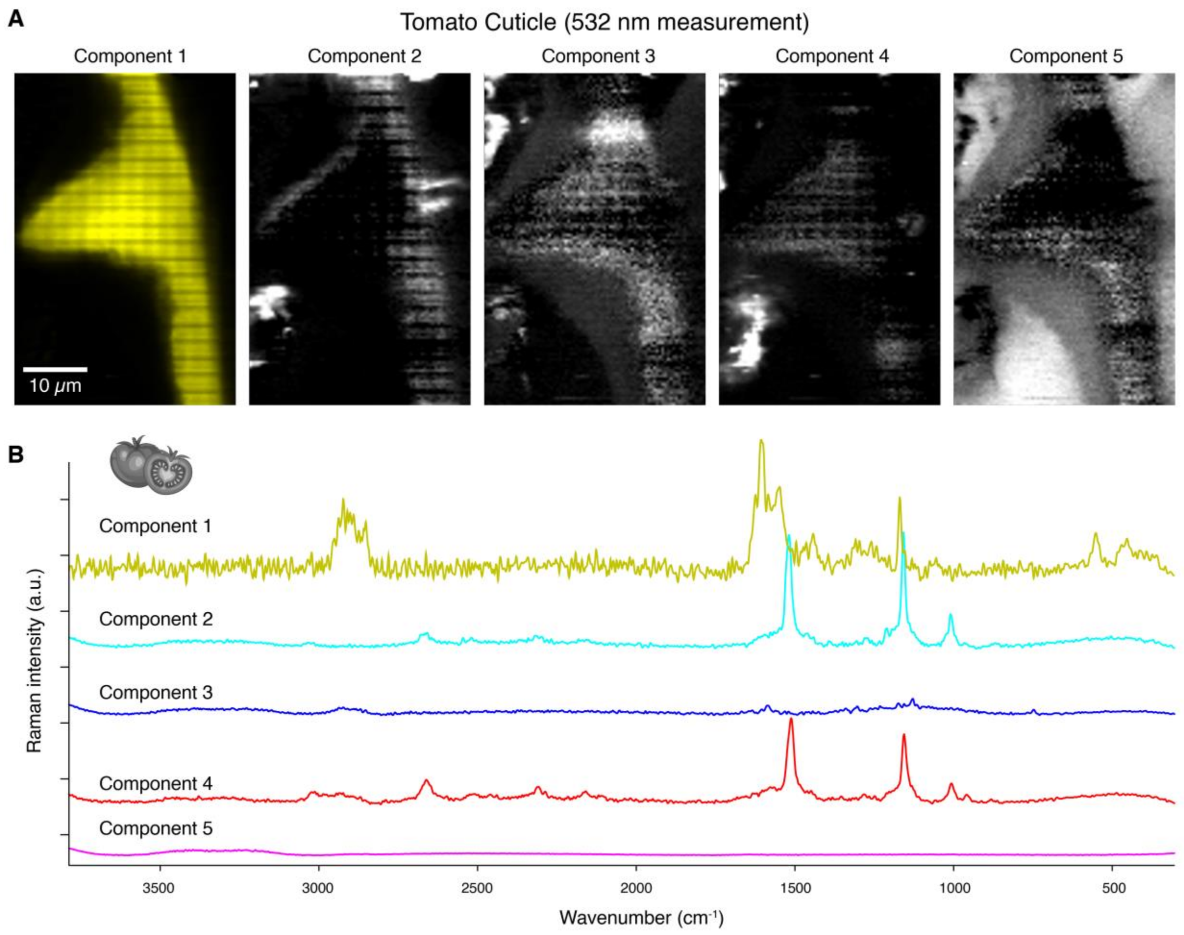

Figure S2. Mixture analysis of a tomato 532 nm Raman measurement. (A) Raman images based on the "True Component Analysis." (B) Corresponding spectra of the different components. Note that the first component spectrum is very noisy and that components two and four and components three and six are very similar. These results demonstrate that the 785 nm laser (Figure 6) is the better choice.
